# Seasonal Variation and Health Risk Assessment of Aflatoxin M1 in Milk From the Central Region of Iran

**DOI:** 10.1002/vms3.71099

**Published:** 2026-07-20

**Authors:** Pedram Hashemi, Mohammadhosein Movassaghghazani, Milica Zekovic

**Affiliations:** ^1^ Faculty of Veterinary Medicine Shab.C. Islamic Azad University Shabestar Iran; ^2^ Department of Food Hygiene and Quality Control Faculty of Veterinary Medicine Shab.C. Islamic Azad University Shabestar Iran; ^3^ Centre of Research Excellence in Nutrition and Metabolism Institute for Medical Research National Institute of Republic of Serbia University of Belgrade Belgrade Serbia

**Keywords:** aflatoxin M1, HPLC, milk, risk assessment, seasonal variation

## Abstract

**Background:**

Aflatoxin M1 (AFM1) contamination in milk is a major public health concern due to its carcinogenicity and resistance to processing.

**Objectives:**

This study assessed seasonal variation in AFM1 levels across raw, pasteurized and ultra‐high‐temperature (UHT) milk and quantified associated non‐carcinogenic and carcinogenic risks.

**Methods:**

A total of 180 samples were collected over four seasons (October 2024–2025), and AFM1 concentrations were measured using HPLC–fluorescence detector (FLD). Distributional characteristics were described using medians and 95th percentile (P95) values, and prevalence estimates were reported with 95% confidence intervals. A health risk assessment was conducted using the estimated daily intake (EDI), hazard index (HI) and margin of exposure (MOE).

**Results and Conclusions:**

AFM1 was detected in all samples (6.10–176.20 ng/kg). Overall, 23.33% (95% CI: 17.7%–30.1%) exceeded the Iranian limit (100 ng/kg), and 58.88% (95% CI: 51.6%–65.8%) surpassed the EU limit (50 ng/kg). Seasonal analysis showed substantially higher contamination during warm months, with raw milk in summer presenting the highest upper tail concentration (P95: 168.57 ng/kg). Risk assessment based on P95 exposure indicated medium non‐carcinogenic risk (HI: 1–10) for children across all milk types and for adults consuming raw milk. Carcinogenic risk characterization showed MOE values below 10000 for children consuming raw and pasteurized milk, indicating inadequate safety margins. These findings demonstrate that reliance on mean AFM1 concentrations underestimates health risks, especially for children. The pronounced seasonal patterns highlight the need for strengthened monitoring and targeted control strategies, with priority given to raw milk during warm seasons to protect vulnerable paediatric populations in Iran.

## Graphical abstract

Seasonal monitoring of milk in central Iran revealed widespread aflatoxin M1 contamination, with significant health risks—especially for children—highlighting the urgent need for targeted control strategies.

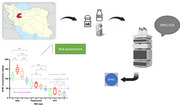

## 1 Introduction

Mycotoxin exposure represents a significant global public health concern, as fungal contamination can affect a wide range of plant‐ and animal‐derived foods under favourable environmental conditions. Although numerous mycotoxins have been identified, only a subset poses major risks to human health, and controlling their occurrence remains challenging. Evidence indicates that mycotoxicosis may be more widespread than previously recognized, particularly in regions with climatic conditions that promote fungal growth. As achieving zero exposure is not feasible, regulatory frameworks aim to keep mycotoxin intake within levels considered tolerable for human health (Singh and Kumari 2022). Aflatoxins are among the most extensively studied mycotoxins due to their strong toxic and carcinogenic effects in humans and animals. Their occurrence is influenced by multiple environmental and agricultural factors, including crop production practices and fungal contamination before harvest, during storage and throughout processing. Because aflatoxins can cause aflatoxicosis and are associated with serious health outcomes, they have received greater scientific and regulatory attention than other mycotoxins (Movassaghghazani and Shabansalmani 2024; Singh and Kumari 2022).

Chronic exposure to low levels of aflatoxins is associated with long‐term health effects, particularly hepatocellular carcinoma (HCC). Aflatoxins are found in a variety of food and feed commodities, and aflatoxin M1 (AFM1)—the hydroxylated metabolite of AFB1—appears in the milk of animals consuming contaminated feed (Singh and Kumari 2022). Contamination of raw milk can lead to the transfer of AFM1 into a wide range of dairy products, including cheese and yogurt, thereby extending consumer exposure beyond milk alone (Bahrami et al. 2016; Mohammadi et al. 2022).

Advances in analytical methods now allow detection of AFM1 at very low concentrations in food samples (Salari et al. 2020). Regulatory limits for AFM1 differ internationally; Iran has established a maximum residue (MRL) limit of 100 ng/kg, whereas the European Union applies a stricter limit of 50 ng/kg (Behtarin and Movassaghghazani 2024). AFM1 is classified by the International Agency for Research on Cancer (IARC) as a Group 1 human carcinogen, with sufficient evidence linking it to HCC (IARC 2012). Mechanistically, aflatoxins exert their carcinogenic effects through metabolic activation to reactive intermediates that form DNA adducts, leading to mutagenesis and liver cancer development (Daou et al. 2020).

The presence of AFM1 in milk and dairy products represents a significant public health concern, particularly in countries with high milk consumption, such as Iran. Periodic monitoring across the production‐to‐consumption chain is therefore essential to protect consumers (Aghebatbinyeganeh et al. 2024; Movassaghghazani and Shabansalmani 2024). Despite the economic and nutritional importance of milk in Iran, studies assessing AFM1 contamination and associated exposure risks remain limited and are often limited to a regional level. The national cancer burden—158 cases per 100000 individuals—and the prominence of liver cancer as a major cause of mortality (IRNA 2018). Underscore the relevance of monitoring carcinogenic contaminants such as AFM1 in staple foods. Global projections indicate a substantial rise in liver cancer incidence and mortality, further highlighting the importance of controlling AFB1 contamination in animal feed, particularly in regions where climatic conditions favour fungal growth (IRNA 2025).

Although several regional studies have investigated AFM1 contamination in milk and dairy products across Iran, comprehensive and seasonally stratified data for Markazi Province remain limited, and quantitative risk assessments for different age groups and milk types are scarce. Therefore, this study aimed to determine the seasonal occurrence and concentration of AFM1 in raw, pasteurized and ultra‐high‐temperature (UHT) cow's milk in Markazi Province and to evaluate the associated non‐carcinogenic and carcinogenic risks for adults and children using estimated daily intake (EDI), hazard index (HI) and margin of exposure (MOE) metrics. By incorporating a percentile‐based analytical framework (P50 and 95th percentile [P95]), this study provides a more accurate characterization of upper tail exposure and seasonal variability, offering improved insight into worst‐case scenarios that may be underestimated by mean‐based approaches. These findings highlight the need for seasonally informed monitoring and regulatory strategies to better protect vulnerable populations, particularly children.

## 2 Materials and Methods

### 2.1 Chemicals and Standards

Sigma‐Aldrich, USA, provided the analytical standard of AFM1 at a concentration of 100 µg/mL in acetonitrile (ACN). Methanol and ACN of HPLC quality were purchased from Merck (Germany).

### 2.2 Sample Collection Method

**FIGURE 1:**
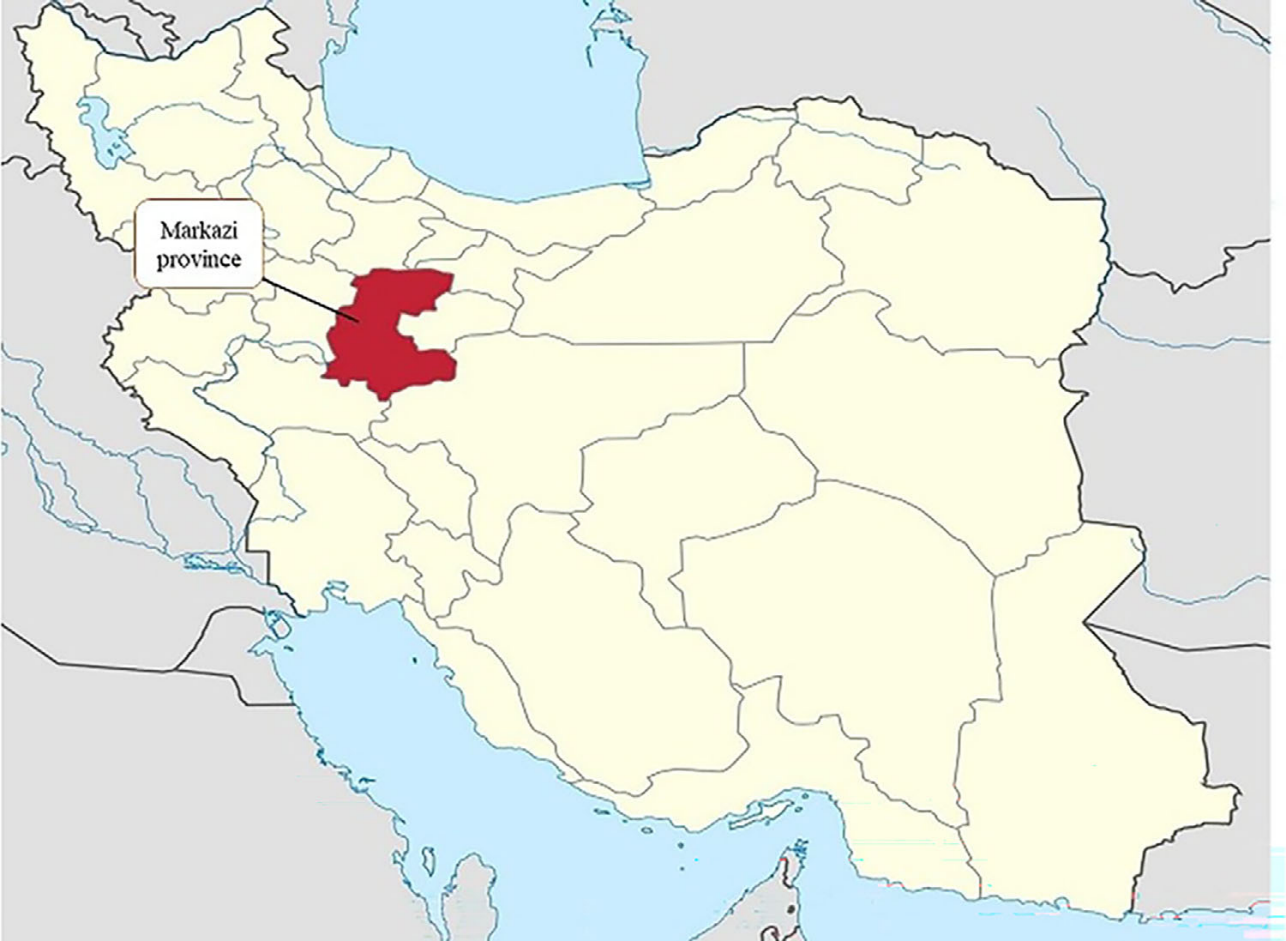
The map of Iran indicates the Markazi Province, the selected region for conducting seasonal milk sampling.

In this study, a total of 180 cow's milk samples were collected using a stratified random sampling approach across four seasons from retail outlets in three cities of Markazi Province (Arak, Khomein and Mahallat) between October 2024 and 2025 (Figure 1). The samples exclusively consisted of cow's milk.

The samples included raw milk (*n* = 60), pasteurized milk (*n* = 60; representing 7 commercial brands) and UHT milk (*n* = 60; representing 12 commercial brands). The retail brands selected included both national and major regional producers. Furthermore, full‐fat products were included to reflect consumer purchasing habits.

To ensure representative coverage of production variability, 15 samples per milk type (raw, pasteurized and UHT) were collected during each season (autumn, winter, spring and summer). All samples were collected in sterile plastic containers and stored at −20°C until further analysis.

### 2.3 Analysis of Samples by HPLC

#### 2.3.1 Sample Preparation

First, 5 mL of milk was mixed with 41 mL of distilled water and centrifuged for 4 min at 5500 rpm and 4°C. Following separation of the fat layer, the aqueous phase was filtered through a pore‐size cellulose acetate filter of 0.45 µm (Millipore, Merck, Germany). Then, 20 mL of filtrate was passed through an immunoaffinity column containing AFM1‐specific antibodies (Waters‐Vicam, USA). After complete binding, the column was washed with distilled water to remove unbound materials. AFM1 was then eluted and concentrated using ACN as the solvent. The eluent was evaporated under a gentle nitrogen stream and reconstituted to a final volume of 1 mL, from which 20 µL was injected into the HPLC system (Behtarin and Movassaghghazani 2024; INSO 2013).

#### 2.3.2 HPLC Conditions

The concentration of AFM1 was determined using an HPLC system (Unicam Crystal‐200 system, England). To conduct the analysis, a reversed‐phase column (ODS‐Column TSK‐Gel TosoHaas) with a length of 25 cm, an internal diameter of 4.6 mm and a particle size of 3 µm was utilized with a fixed temperature of 30°C. ACN, methanol and water were the components taken in a volumetric composition of 17:23:60 to prepare the mobile phase, which was delivered through the column with a flow rate of 1.1 mL/min and a pressure of 2900 psi.

Detection was performed using a fluorescence detector (FLD) set at an excitation wavelength of 362 nm and an emission wavelength of 435 nm. Following sample injection, the peak area corresponding to the retention time was measured and quantified against a calibration curve constructed using the AFM1 standard. The retention time (*R_t_*) for AFM1 under these chromatographic conditions was determined to be 11.9 min. The retention time was stable across runs.

### 2.4 Method Validation

Quantification of AFM1 in samples required the construction of a robust calibration curve utilizing AFM1 standards prepared across six concentration levels (0.00–200 ng/kg) in an ACN/water mixture containing 0.2% of HCOOH (1/1, v/v), with each concentration analysed in triplicate. Sample concentrations were determined on the basis of the linear relationship established between standard peak areas and their respective concentrations, with the overall linearity confirmed via the coefficient of determination (*r* ^2^) from the residual plot of the calibration curve. The analytical limits, limit of detection (LOD) and limit of quantitation (LOQ), were set at signal‐to‐noise (S/N) ratios of 3:1 and 10:1, respectively. Method specificity was ensured by comparing retention times in blank matrix samples, using a spiking level of 50 ng/kg of AFM1 to prevent potential interference with the target analyte's retention time. Matrix effects were evaluated across three matrices (raw, pasteurized and UHT milk) by comparing the slopes of solvent‐based (*b*_sol) and matrix‐matched (*b*_mm) calibration curves, yielding the signal suppression/enhancement (SSE) ratio as per Equation (1). Finally, method precision, expressed as relative standard deviation (RSD), was determined through comprehensive intra‐day and inter‐day repeatability tests conducted on spiked samples fortified at three levels: 40, 80 and 120 ng/kg (INSO 2013; Rabie et al. 2024):

(1) $$\mathrm{SSE}\;(\%)=\frac{b_{\mathrm{mm}}-b_{\mathrm{sol}}}{b_{\mathrm{sol}}}\;\times 100$$

**FIGURE 2:**
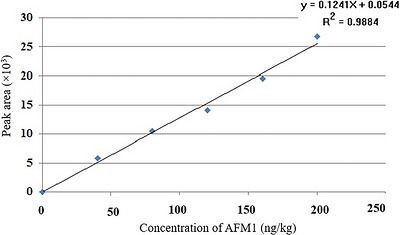
Calibration curve for the quantification of AM1 by HPLC, demonstrating linearity with a coefficient of determination *r* ^2^ = 0.9884. AFM1, aflatoxin M1.

Figure 2 presents the calibration curve utilized for the quantification of AFM1 in the tested milk samples. The curve exhibits strong linearity across the working range, evidenced by a high coefficient of determination (*r* ^2^ = 0.9884), thereby confirming the reliability of the method's response. This value meets the performance criteria recommended by the Iranian National Standards Organization (INSO 2013).

Table 1 presents the method validation parameters established for the determination of AFM1 in various milk matrices (raw, pasteurized and UHT) using HPLC. Method validation ensures the accuracy, precision and reliability of the analytical technique across different sample types and concentration ranges. Key validation metrics, such as precision (measured by intra‐day and inter‐day RSD), accuracy (measured by extraction recovery, ER), matrix effects (evaluated by SSE), LOD and LOQ, are reported at multiple spiked concentration levels.

**TABLE 1:** Validation parameters for determining aflatoxin M1 (AFM1) in milk samples by the HPLC method.

Milk samples	Spiked level (ng/kg)	Intra‐day (*n* = 3) RSD^a^ (%)	Inter‐day (*n* = 4) RSD^a^ (%)	ER (%)	Matrix effect SSE^b^ (%)	LOD^c^ (ng/kg)	LOQ^d^ (ng/kg)
Raw	40	3.17	6.07	97.51	−2.16	0.926	2.726
	80	5.40	3.18	104.12			
	120	4.30	2.84	92.27			
Pasteurized	40	5.71	3.51	96.84	3.58	1.341	3.210
	80	6.36	5.06	88.92			
	120	4.02	4.70	93.33			
UHT	40	3.93	5.70	87.65	5.09	0.659	1.838
	80	2.76	3.30	105.04			
	120	4.65	6.69	95.35			

Abbreviations: ER, extraction recovery; LOD, limit of detection; LOQ, limit of quantitation; RSD, relative standard deviation; SSE, signal suppression/enhancement; UHT, ultra‐high‐temperature.

^a^Relative standard deviation for intra‐ and inter‐day precisions at concentrations of 40,80 and 120 ng/kg of AFM1.

^b^Signal suppression/enhancement—The SSE value reflects the average slope ratio across spiked levels for each matrix (raw, pasteurized and UHT).

^c^Limit of detection (S/N = 3).

^d^Limit of quantification (S/N = 10).

The validation results in Table 1 confirm that the developed HPLC method is highly suitable and reliable for the accurate and precise determination of AFM1 across raw, pasteurized and UHT milk matrices. The precision data, indicated by RSD values consistently below 7% for both intra‐day and inter‐day measurements, demonstrate excellent repeatability and reproducibility. Accuracy was also confirmed by acceptable ER values, ranging from 87.65% to 104.12%, which fall within the required limits for mycotoxin analysis. The recovery rate was calculated from matrix‐matched spiked samples. Crucially, the method exhibits high sensitivity, with a low LOQ between 1.838 and 3.210 ng/kg; this ensures quantitative accuracy well below both EU and Iranian MRL, which strengthens the credibility of the findings. Finally, the minimal observed matrix effects indicate that the sample preparation effectively manages matrix interference, ensuring the robustness of the quantitative results.

### 2.5 Risk Evaluation

Because all AFM1 values were above the LOQ, the lower bound (LB), middle‐bound (MB) and upper bound (UB) scenarios produced identical results. For this reason, only one set of exposure estimates is presented.

#### 2.5.1 Daily Exposure Level

For milk consumers, the EDI or dietary exposure to AFM1 was calculated using Equation (2) (Aghebatbinyeganeh et al. 2024). A daily milk consumption of 0.192 kg/day per person has been assumed for the Iranian population. The standard body weight used for risk assessment, based on FAO/WHO guidelines, is 70 kg for adults and 15 kg for children (Behtarin and Movassaghghazani 2024):

(2) $$\mathrm{EDI}\left(\mathrm{ng}/\mathrm{kg}\;\mathrm{bw}/\mathrm{day}\right)=\frac{C\times D}{\mathrm{bw}\;}$$

where *C* is the concentration of AFM1 in milk (ng/kg), *D* is the daily milk consumption (kg/day), and bw is the average body weight (kg).

#### 2.5.2 The HI

The HI was calculated by dividing the EDI by the threshold dose (TD50) of aflatoxin, which is 10.4 µg/kg body weight (bw)/day, and subsequently dividing the result by an uncertainty factor of 50000, according to Equation (3). At the TD50 dose, 50% of the test animals developed tumours. The tolerable daily intake (TDI) was derived by dividing TD50 by 50,000, resulting in a TDI value of 0.2 ng/kg body weight/day for AFM1. The HI was then determined based on this calculation (Kuiper‐Goodman 1990; Aghebatbinyeganeh et al. 2024; Behtarin and Movassaghghazani 2024):

(3) $$\mathrm{HI}=\frac{\mathrm{EDI}}{\mathrm{TD}50/50000}$$

#### 2.5.3 The MOE

MOE was utilized to characterize the potential public health risk from AFM1 exposure through the consumption of various milk categories within Markazi Province. This calculation was performed by taking the ratio of the benchmark dose lower confidence limit (BMDL10) to the EDI, expressed by Equation (4). In accordance with the established guidance provided by the European Food Safety Authority (EFSA) for this potent carcinogen, a conservative BMDL10 value of 4 µg/kg bw/day was adopted for the AFM1 risk assessment, thereby enabling the determination of risk‐based prioritization (Schrenk et al. 2020):

(4) $$\mathrm{MOE}=\frac{\mathrm{BMDL}10}{\mathrm{EDI}}$$

### 2.6 Data Analysis Method

Normality of AFM1 concentration data was evaluated using the Shapiro–Wilk test. The results indicated that several groups deviated significantly from normality (*p* < 0.05). Therefore, AFM1 concentrations were summarized using the median, interquartile range (IQR) and the P95, which better represent the skewed distribution of the data. Mean and standard deviation were additionally reported for descriptive completeness but were not used for inferential comparisons. Statistical analyses were performed using IBM SPSS Statistics (version 27). Differences in AFM1 concentrations across seasons and milk types were assessed using the Kruskal–Wallis test due to the non‐normal distribution of the data. When significant differences were detected, Dunn's post hoc test with Bonferroni adjustment was applied. For datasets that met normality assumptions, one‐way analysis of variance (ANOVA) followed by Tukey's post hoc test was used. To visualize distributional patterns and highlight seasonal shifts in the upper tail of contamination, grouped box‐and‐whisker plots were generated using GraphPad Prism (version 10.2.0). All statistical tests were two‐tailed, and significance was set at *p* < 0.05.

## 3 Results

### 3.1 The Mean of AFM1 in Milk Samples

**FIGURE 3:**
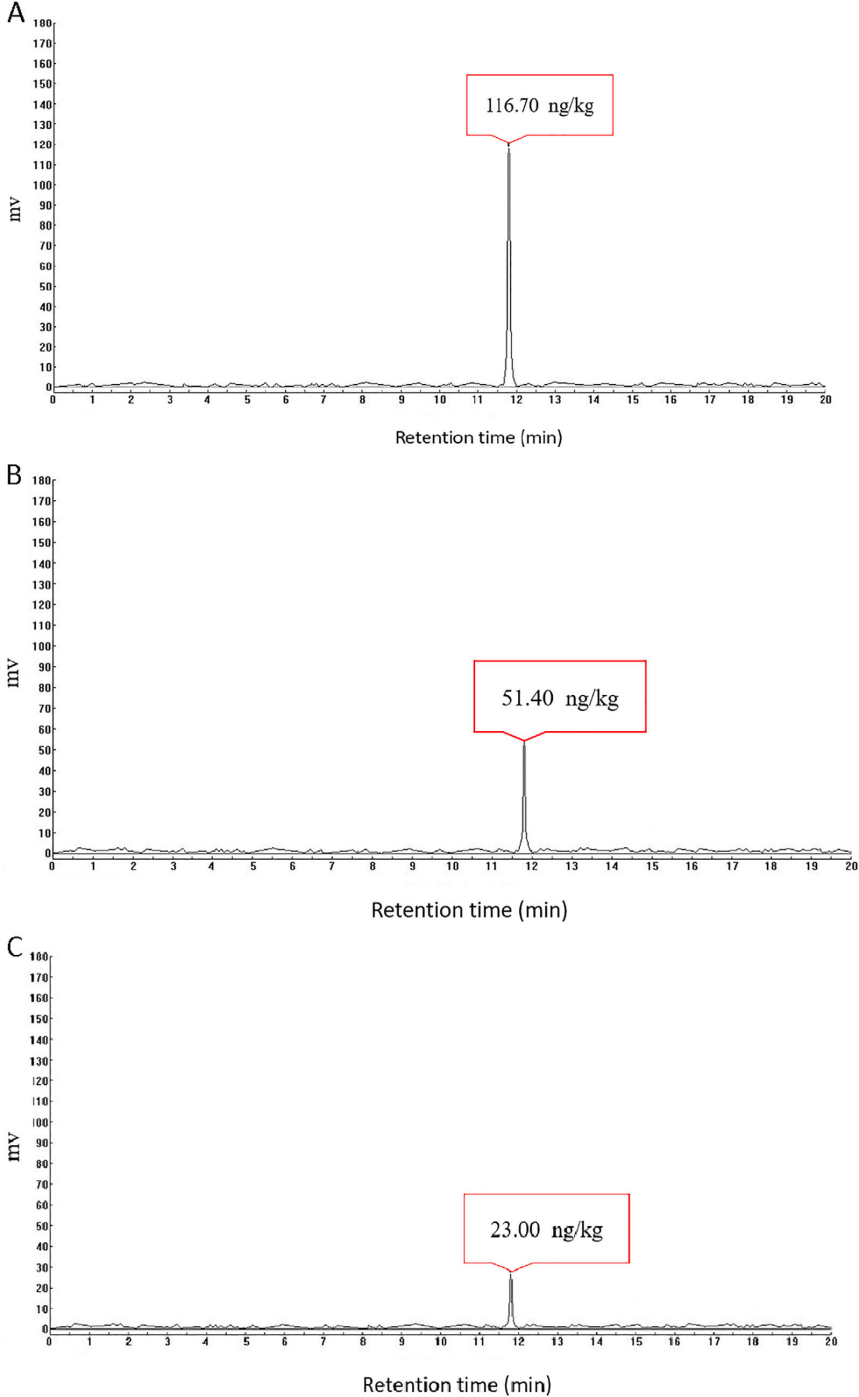
Representative HPLC chromatograms of AFM1 in raw (A), pasteurized (B) and UHT milk (C).

The chromatograms corresponding to raw milk, pasteurized milk and UHT milk samples are presented in Figure 3.

As shown in Table 2, although a significant portion of raw milk samples exceeded both standards, the 95% CI reveals the statistical range of prevalence. Notably, in raw milk during summer, the prevalence of samples exceeding the INSO limit was 100% (95% CI: 79.6%–100%). In contrast, UHT milk showed the highest compliance, with a 0% (95% CI: 0.0%–19.3%) exceeding rate for the INSO limit across all seasons.

**TABLE 2:** Distribution‐based descriptive statistics of aflatoxin M1 (AFM1) concentrations (ng/kg) in raw, pasteurized and ultra‐high‐temperature (UHT) milk samples across four seasons in Markazi Province, Iran.

									Number of positive samples (exceeding maximum residue limit) % [95% CI]		Range of AFM1 Min–Max	
Milk type	Season	Number of samples	Median (P50)	IQR (Q1–Q3)	P75	P90	P95	Mean ± SD	Iranian standard >100 ng/kg	EU standard >50 ng/kg	Min	Max
Raw	Spring	15	116.70	103.2–123.2	123.20	127.86	130.51	113.31 ± 13.67	12 (80.0) [54.8, 93.0]	15 (100.0) [79.6, 100.0]	84.70	132.40
	Summer	15	145.30	135.75–156.8	156.8	162.82	168.57	146.29 ± 15.05	15 (100.0) [79.6, 100.0]	15 (100.0) [79.6, 100.0]	124.10	176.20
	Autumn	15	112.50	106.65–136.95	136.95	145.68	151.13	119.75 ± 19.39	13 (86.7) [62.1, 96.3]	15 (100.0) [79.6, 100.0]	96.20	157.50
	Winter	15	89.40	83–95.7	95.7	101.26	104.51	89.05 ± 10.49	2 (13.3) [3.7, 37.9]	15 (100.0) [79.6, 100.0]	70.80	107.80
	All seasons	60	115.25	97.92–135.75	135.75	152.58	157.58	117.10 ± 25.21	42 (70.0) [58.4, 81.6]	60 (100.0) [94.0, 100.0]	70.80	176.20
Pasteurized	Spring	15	63.10	58.5–84.35	84.35	89.78	91.62	69.12 ± 15.36	0 (0.0) [0.0, 19.3]	14 (93.3) [70.2, 98.8]	48.30	94.70
	Summer	15	75.20	58.8–79.45	79.45	82.98	83.93	70.66 ± 12.01	0 (0.0) [0.0, 19.3]	14 (93.3) [70.2, 98.8]	49.70	85.40
	Autumn	15	61.20	53.65–67.40	67.40	70.00	71.27	60.32 ± 10.05	0 (0.0) [0.0, 19.3]	12 (80.0) [54.8, 93.0]	37.40	73.30
	Winter	15	46.60	40.65–49.15	49.15	56.38	58.57	46.52 ± 7.05	0 (0.0) [0.0, 19.3]	3 (20.0) [7.0, 45.2]	37.70	60.60
	All seasons	60	60.80	49.57–71.12	71.12	82.94	85.96	61.65 ± 14.81	0 (0.0) [0.0, 5.9]	43 (71.7) [60.3, 83.1]	37.40	94.70
UHT	Spring	15	46.6	12.35–49.15	45.40	46.56	47.98	32.71 ± 16.41	0 (0.0) [0.0, 19.3]	1 (6.7) [1.2, 29.8]	8.00	51.20
	Summer	15	47.40	21.20–49.30	49.30	50.94	53.35	38.78 ± 15.28	0 (0.0) [0.0, 19.3]	2 (13.3) [3.7, 37.9]	16.10	57.20
	Autumn	15	34.10	16.45–36.30	36.30	38.28	39.34	28.79 ± 10.35	0 (0.0) [0.0, 19.3]	0 (0.0) [0.0, 19.3]	13.10	40.60
	Winter	15	25.50	9.80–28.15	28.15	31.48	33.05	21.36 ± 10.18	0 (0.0) [0.0, 19.3]	0 (0.0) [0.0, 19.3]	6.10	35.50
	All seasons	60	32.55	16.40–42.85	42.85	49.21	49.87	30.41 ± 14.49	0 (0.0) [0.0, 5.9]	3 (5.0) [0.0, 10.5]	6.10	57.20
Total	—	180	60.80	39.6–96.77	96.77	129.97	145.45	69.72 ± 40.53	42 (23.3) [17.7, 30.1]	106 (58.88) [51.6, 65.8]	6.10–176.20	

*Note*: The Iranian standard limit—milk <100 ng/kg. The European standard—milk <50 ng/kg. [95% CI]: 95% confidence interval calculated using the Wilson score method.

Abbreviations: SD, standard deviation; IQR, interquartile range.

Among the total samples collected, 23.33% (95% CI: 17.7%–30.1%) exceeded the maximum permissible limit established by Iranian standards, whereas 58.88% (95% CI: 51.6%–65.8%) surpassed the regulatory limit set by the EU.

**FIGURE 4:**
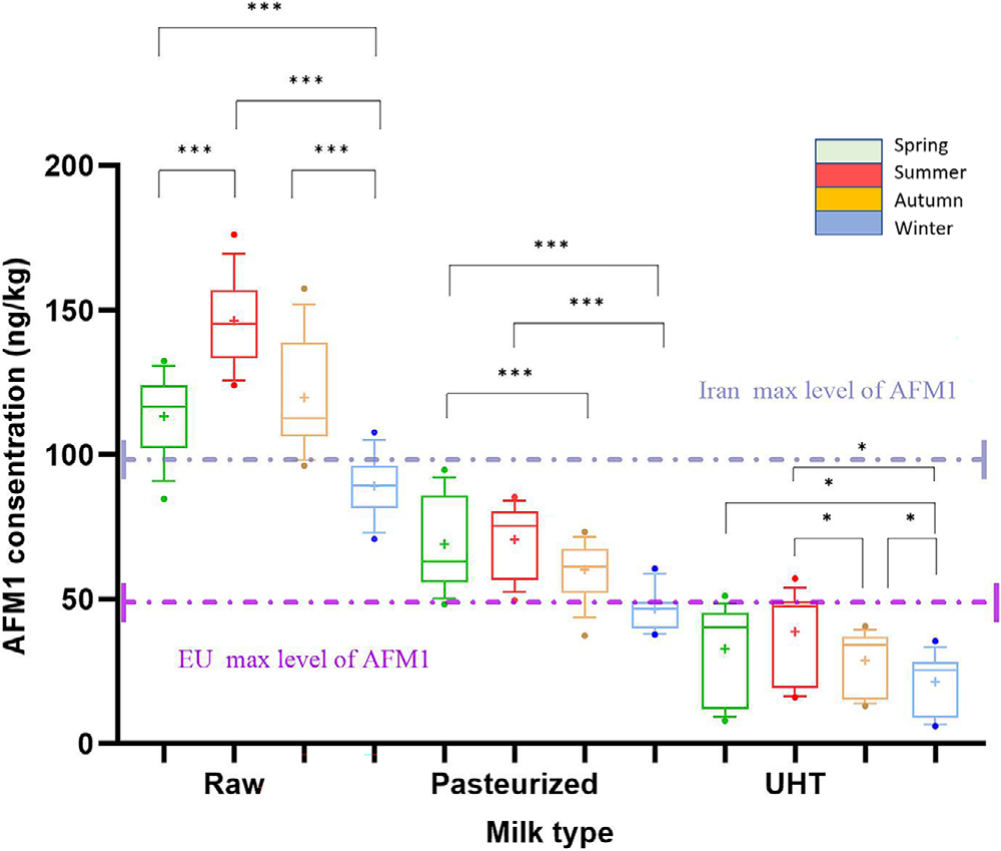
Seasonal variation of AFM1 concentration in different milk types in Markazi Province, Iran. Boxplots display the median and interquartile range (25th–75th percentiles), with whiskers representing the 10th–90th percentiles. Mean values are shown as ‘+’ symbols. Statistical differences in AFM1 concentration among milk types are indicated by *p < 0.05 and ***p < 0.001. Comparisons for raw and pasteurized milk were performed using one‐way ANOVA followed by Tukey's post hoc test, whereas UHT milk was analysed using the Kruskal–Wallis test followed by Dunn's post hoc test. AFM1, aflatoxin M1.

A comparison of AFM1 levels across the four seasons in different milk types is illustrated in Figure 4. According to the boxplot distributions, raw milk exhibited the highest AFM1 concentrations during summer, whereas pasteurized milk showed elevated levels in both spring and summer. For UHT milk, a statistically significant difference was observed only between summer and winter (adjusted *p* value = 0.0017), whereas other seasonal comparisons showed numerical but non‐significant differences after adjustment.

In raw milk, AFM1 concentrations differed significantly across seasons, indicating a clear seasonal pattern. In pasteurized milk, although no significant difference was detected between spring and summer, both seasons showed significantly higher AFM1 levels compared with autumn and winter. For UHT milk, only the summer–winter comparison reached statistical significance.

These findings suggest that environmental or seasonal factors—particularly those prevalent in summer—may contribute to increased AFM1 contamination, whereas winter conditions are associated with lower toxin levels. Boxplots display the median, IQR (25th–75th percentiles) and 10th–90th percentile whiskers, with mean values.

**FIGURE 5:**
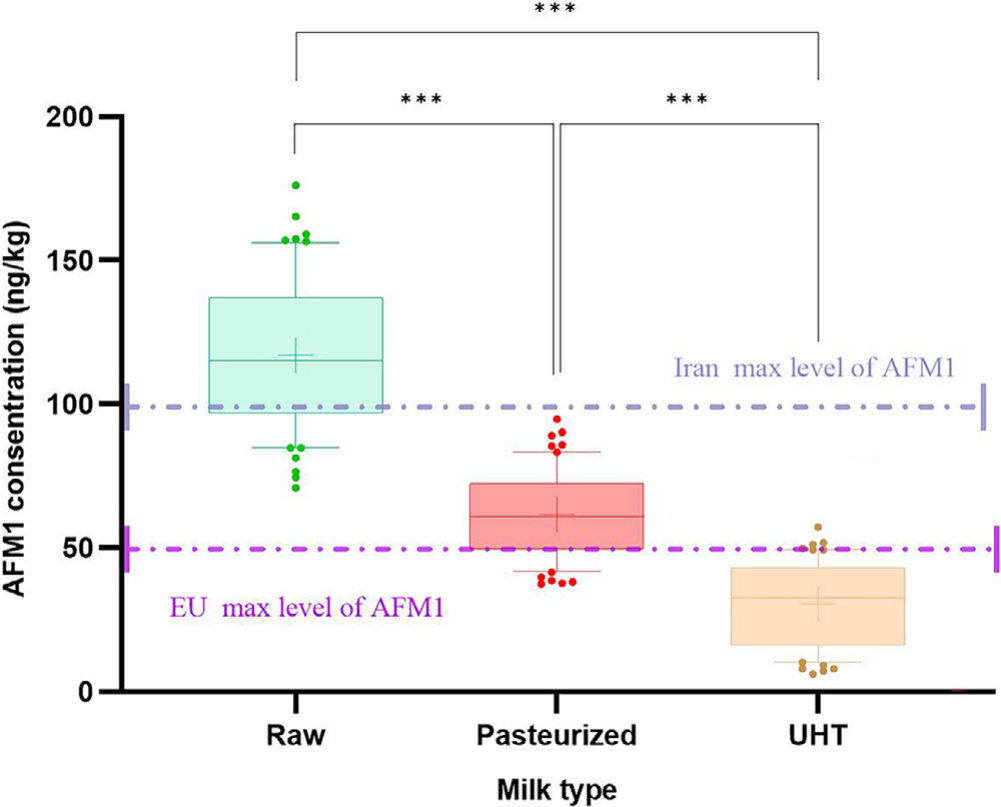
Variation of AFM1 concentration among different milk types in Markazi Province, Iran. Boxplots display the median and interquartile range (25th–75th percentiles), with whiskers representing the 10th–90th percentiles. Mean values are shown as ‘+’ symbols. Statistical differences among milk types are indicated by ***p < 0.001, based on one‐way ANOVA followed by Tukey's post hoc test. UHT, ultra‐high‐temperature.

Figure 5 presents the distribution of AFM1 concentrations across the three milk types in Markazi Province, Iran. According to the boxplot results, raw milk exhibited the highest AFM1 concentrations, whereas UHT milk showed the lowest levels. Based on the four‐season dataset, 23.33% (95% CI: 17.7%–30.1%) of all analysed samples exceeded the Iranian MRL. The highest AFM1 levels were observed in raw milk, whereas UHT milk showed the lowest concentrations. Seasonal patterns were evident: Raw milk showed elevated AFM1 concentrations during summer, pasteurized milk exhibited higher levels in spring and summer, and UHT milk showed increased concentrations in spring, summer and autumn. These findings indicate a clear seasonal influence on AFM1 contamination, with summer consistently associated with higher concentrations. Boxplots display the median, IQR (25th–75th percentiles) and 10th–90th percentile whiskers, with mean values.

### 3.2 Risk Assessment of AFM1 Exposure

Table 3 presents a distribution‐based assessment of AFM1 exposure in various milk types for adults and children. Adults’ EDI values remain relatively low across all circumstances, with HI values frequently below or slightly above 1. Raw milk poses a low‐to‐moderate non‐carcinogenic risk, with an HI of 2.16 in the P95 scenario. Adults continue to have MOE values over the EFSA safety threshold of 10000 for pasteurized and UHT milk, whereas raw milk has a borderline concern at P95 (MOE = 9300), indicating a small but not negligible carcinogenic risk at high‐exposure settings.

**TABLE 3:** Estimated daily intake (EDI), hazard index (HI) and margin of exposure (MOE) for aflatoxin M1 (AFM1) through consumption of different milk types among adults and children.

Target group	Exposure scenario	Parameter	Raw milk	Pasteurized milk	UHT milk
Adults (70 kg)	Median (P50)	EDI (ng/kg bw/day)	0.316	0.167	0.089
		HI	1.58	0.83	0.45
		MOE	12,700	24,000	44,900
	Upper quartile (P75)	EDI (ng/kg bw/day)	0.372	0.195	0.118
		HI	1.86	0.98	0.59
		MOE	10,800	20,500	340,000
	High exposure (P95)	EDI (ng/kg bw/day)	0.432	0.236	0.137
		HI	2.16	1.18	0.68
		MOE	9300	17,000	29,000
Children (15 kg)	Median (P50)	EDI (ng/kg bw/day)	1.48	0.78	0.42
		HI	7.38	3.89	2.08
		MOE	2700	5100	9600
	Upper quartile (P75)	EDI (ng/kg bw/day)	1.74	0.91	0.55
		HI	8.69	4.55	2.74
		MOE	2300	4400	7300
	High exposure (P95)	EDI (ng/kg bw/day)	2.02	1.10	0.64
		HI	10.09	5.50	3.19
		MOE	2000	3600	6300

*Note*: All exposure and risk values (EDI, HI and MOE) are based on rounded calculations. HI = hazard index (<1 low risk, 1–10 medium risk and >10 high risk). MOE (<10000 serious health concern, >10000 low health concern) according to EFSA guidance. Adults (18–70 years). Children (3–10 years). P50: 50th percentile. P75: 75th percentile. P95: 95th percentile.

Abbreviation: bw, body weight.

In contrast, children have much higher exposure and risk levels for all milk kinds. Children's EDI levels are four to five times greater than adults’ because they have a lower body weight and consume more milk per kilogram of body weight. Children are the most susceptible group, with HI values over 1 in all scenarios and milk types, peaking at 10.09 for raw milk at P95. This indicates a medium non‐carcinogenic risk. MOE values for children are continuously less than 10000, even at the median (P50) level for raw and pasteurized milk, indicating insufficient safety margins and a potential carcinogenic risk. UHT milk has a reduced risk but remains below the MOE level in all circumstances.

The table reveals that children encounter both non‐carcinogenic and carcinogenic health hazards from all milk types, but adults face relatively minimal concerns, primarily from raw milk. These findings highlight the importance of focused risk management measures, especially for paediatric populations.

## 4 Discussion

### 4.1 The Mean of AFM1 in Milk Samples

These findings from the present study are consistent with those reported by Aghebatbinyeganeh et al. (2024), who observed higher AFM1 concentrations in raw milk during the summer season in the Lorestan and Ilam provinces of Iran compared to other seasons (Aghebatbinyeganeh et al. 2024). The elevated AFM1 levels observed in summer are consistent with the known increase in AFB1 production by *Aspergillus* spp. under warm and humid conditions. The mould contaminates animal feed commodities (e.g., grains and meals). Livestock consuming this contaminated feed convert the ingested AFB1 into AFM1 through biotransformation, resulting in the excretion of AFM1 via milk.

It appears that climate variations in different regions of Iran affect the level of contamination of animal feed with mycotoxins. Increased temperatures lead to higher fungal contamination in animal feed and increased levels of AFB1, and consumption of contaminated forage by livestock increases the AFM1 levels in milk. For the growth of aflatoxin‐producing moulds, temperatures between 25°C and 37°C and humidity levels of 80%–85% are necessary (Bilandžić et al. 2022). Detailed local climatic data (such as temperature and humidity records) were not incorporated into the present analysis, which limits the direct quantitative attribution of the observed seasonal AFM1 variation to specific environmental patterns. However, in recent years, increased temperatures during spring and summer have been noticeable in various regions of Iran. The temperature and humidity conditions in Markazi Province facilitate the growth of aflatoxin‐producing moulds.

In a study by Shabansalmani and Movassaghghazani, AFM1 levels were examined in milk and dairy products in Tehran. In total, 75 samples—including raw milk, pasteurized milk, UHT milk, pasteurized yogurt and pasteurized cheese—were collected. The AFM1 levels in all samples were higher than the European Union's permissible limit. Additionally, the AFM1 levels in all milk samples also exceeded the maximum permitted level in Iran (Shabansalmani and Movassaghghazani 2023). In the present study, 23.33% (95% CI: 17.7%–30.1%) of the milk samples contained AFM1 above the permissible limit in Iran.

In the study by Behtarin and Movassaghghazani (2024), AFM1 was detected in all dairy samples collected in Tabriz, and all levels were below the Iranian permissible limit. Similar to the present study, UHT milk showed the lowest AFM1 concentration, likely reflecting the use of higher quality raw milk from industrial dairies in UHT production.

Hasninia et al. reported a much lower exceedance rate of AFM1 in Kermanshah (16.7%) compared with the markedly higher level observed in Markazi (58.88%). This contrast points to regional differences in feed quality and environmental conditions rather than seasonal effects alone (Hasninia et al. 2022).

In the study by Ghaffarian‐Bahraman et al. (2023), AFM1 contamination in milk from Kerman and Rafsanjan was generally low, with only 20% of samples exceeding the EU limit. In contrast, the higher exceedance rate observed in Markazi Province suggests that differences in climatic conditions between the two regions—particularly the hot and arid environment of Kerman compared with the more temperate climate of Markazi—may contribute to the discrepancy in AFM1 levels.

In Isfahan, Jafari et al. (2021) reported that 44.87% of raw and pasteurized milk samples exceeded the EU limit, with mean AFM1 levels lower than those observed in the present study.

Similarly, Mokhtari et al. (2022) found AFM1 concentrations ranging from 57 to 270 ng/L in northwestern Iran, with higher levels in pasteurized and UHT milk than in raw milk, a pattern that differs from our findings. In Golestan Province, AFM1 was detected in all milk types, with the highest levels in raw cow's milk; however, except for UHT milk, AFM1 concentrations in raw and pasteurized milk were still lower than those measured in Arak (Jorjani and Movassaghghazani 2023). Together, these studies highlight notable regional variability in AFM1 contamination across Iran.

Nemati et al. determined AFM1 levels in 84 raw milk samples by chromatography in Ilam province. All samples (100%) contained AFM1. Only 2.38% of the samples had AFM1 above the Iranian permissible limit (Nemati et al. 2025). The results of the present study did not correspond with the study conducted in Ilam, as the AFM1 level in raw milk samples in Markazi Province was higher.

Results of a study in Fasa city, Fars province, Iran, showed that out of 180 samples of milk and dairy products collected during hot and cold seasons, 85.55% of samples contained AFM1. 68.88% of samples had AFM1 levels above the permissible limit of Iran and the EU. The range of AFM1 levels in the samples was 1.10–453.50 ng/kg (Heidari et al. 2024). In the present study, the AFM1 range was lower than in Fasa city.

In the study by Mofid et al. (2024), AFM1 was detected in 61% of pasteurized milk samples collected from several Iranian cities, with only 9.26% exceeding the national limit. This proportion is considerably lower than what was observed in the present study. However, these differences should be interpreted cautiously, given the limited number of samples per city and the wide climatic variation across the surveyed regions.

In Yazd Province, Fallah et al. reported AFM1 contamination in cow, sheep, goat and camel milk, with exceedance rates generally lower than those observed in the present study. AFM1 levels were higher in milk from traditional farms, and seasonal variation influenced both occurrence and concentration (Fallah et al. 2016). In contrast, in the present four‐season dataset, AFM1 was detected in all samples, and the proportion exceeding the legal limit was notably higher than in the Yazd study.

The findings of the present study in Markazi Province align with, yet also differ from, the national patterns reported in the systematic review and meta‐analysis by Khaneghahi Abyaneh et al. (2020). Their pooled mean AFM1 concentration in Iranian milk was 55.97 ng/kg, with substantial variability across analytical methods and regions, and the highest contamination was observed in northern and humid areas. In comparison, the AFM1 levels detected in Markazi Province were generally lower than the national pooled estimates for UHT milk (94.81 ng/kg) and closer to the reported means for raw and pasteurized milk (55.08 and 49.76 ng/kg, respectively). Both studies highlight seasonal variation, with the meta‐analysis identifying winter as the season with the highest AFM1 levels, which is consistent with the seasonal elevation observed in our dataset. These comparisons suggest that whereas Markazi Province does not represent a high‐contamination hotspot relative to northern Iran, seasonal shifts and feed‐related factors still play a critical role in shaping AFM1 exposure patterns in the region.

In a study conducted in Croatia, the seasonal levels of AFM1 in cow's milk were determined between winter 2016 and winter 2022. Overall, 5817 cow milk samples were screened for AFM1 concentration using the ELISA method. To confirm AFM1 concentrations above the MRL set by the European Union, ultra‐high‐performance liquid chromatography coupled with tandem mass spectrometry was used. In 94.7% of the milk samples, the AFM1 level was below the ELISA's LOD. In 3.47% of samples, AFM1 levels were between the detection limit and the MRL. Only 1.87% of all samples exceeded the MRL. The mean increased values of AFM1 in different seasons ranged from 59.2 ng/kg (autumn 2017) to 387.8 ng/kg (autumn 2021). The highest prevalence of positive AFM1 samples was observed in the autumn and winter, with a maximum (6.4%) in winter 2019/2020 (Bilandžić et al. 2022). The results of the present study did not match those of the study in Croatia, one reason for which may be differences in climatic conditions.

In a study by Hattimare et al., the level of AFM1 in 146 samples of milk and dairy products from retail markets in Chhattisgarh, India, was determined using high‐performance liquid chromatography and an FLD. In total, 52 samples (35.6%) contained AFM1, with overall concentrations ranging from undetectable to 2.608 µg/L. AFM1 concentrations exceeding the maximum permissible level set by the European Commission were observed in 94.2% of the positive samples (Hattimare et al. 2022).

In the study conducted in Lebanon, AFM1 contamination was reported across raw, pasteurized and UHT milk, with 28%, 54.5% and 45.5% of samples exceeding the EU limit (Daou et al. 2020). In comparison, the exceedance rates observed in Arak Province were higher for raw and pasteurized milk but lower for UHT milk. Although AFM1 levels in Arak were within a broader range than those reported in Lebanon, the findings, however, indicate substantial regional variations in contamination patterns.

Iqbal et al. collected a total of 372 milk and dairy product samples (169 in summer, 203 in winter) from major cities in Punjab, Pakistan (2014–2015), and analysed for AFM1 using HPLC–FLD. AFM1 was detected in 45.5% of summer and 56.1% of winter samples, with concentrations ranging up to 229.6 and 345.8 ng/L, respectively. Raw milk had the highest mean AFM1 levels: 94.9 ± 5.4 ng/L in summer and 129.6 ± 8.4 ng/L in winter. A significant proportion of samples exceeded the EU limit of 50 ng/L, particularly in winter (Iqbal et al. 2017). The findings of the study conducted in Pakistan were consistent with the present study, only with respect to elevated levels of AFM1 in raw milk.

Xiong et al. assessed AFM1 contamination in raw milk from 18 dairy farms in China's Yangtze River Delta across four seasons. A total of 72 samples were analysed using LC–MS/MS, with AFM1 detected in 59.7% of samples (10–420 ng/L). AFM1 levels were significantly higher in winter (123 ng/L) compared to other seasons, which showed no significant differences. The findings highlight winter as a high‐risk period for AFM1 contamination, emphasizing the need for seasonal management of aflatoxins in feed and milk (Xiong et al. 2013). The results of the study in China did not align with the findings of the present dataset.

The ELISA and HPLC are the standard techniques used for detecting AFM1. Although the ELISA method is widely recognized for being inexpensive, readily available and rapid, the HPLC technique is ultimately more highly valued for its superior accuracy and reliability in analysis (Movassaghghazani and Shabansalmani 2024). On the basis of the preceding discussion regarding aflatoxin measurement techniques, the variance in the toxin levels reported across different studies can, in some instances, be attributed to the specific measurement method and its inherent precision. This is highly relevant, as techniques like HPLC are known to offer high analytical accuracy.

Effective options for reducing AFM1 contamination in the dairy supply chain include increasing animal feed monitoring, particularly for AFB1‐producing fungus, as well as improving feed storage and handling practices year‐round. Transitioning from traditional to more controlled industrial farming systems can also help reduce exposure because these systems often have stricter feed quality management. Effective year‐round feed management is crucial for minimizing fungal growth and aflatoxin generation, particularly during cold seasons when animals rely largely on stored feed.

### 4.2 Risk Assessment of AFM1 Exposure

Because of their lower body weight and proportionately higher intake in relation to body mass, children consistently show a higher risk across all milk categories. UHT milk has the lowest risk profile, whereas pasteurized milk and raw milk are the most concerning. However, children's exposure to UHT milk surpasses the TDI and results in MOE values below the suggested threshold, suggesting that no milk type is completely risk‐free for this vulnerable population.

Risk assessment in the study by Behtarin and Movassaghghazani (2024) in Tabriz indicated that the HI for all samples was less than one for both adult and child consumers, except for milk samples for children, which were greater than one, indicating a moderate risk.

The results of the study by Aghebatbinyeganeh et al. (2024) in the provinces of Ilam and Lorestan showed that the definitive risk assessment of milk and dairy consumption does not pose a risk for liver cancer in adults, but it did indicate significant concerns for children who consume milk and yogurt (HI > 1). The findings from Ilam and Lorestan matched those from Arak province.

In Lebanon, AFM1 consumption was associated with 0.0041 additional cases of cancer per 100000 people annually. On the basis of these findings, milk and dairy consumption in Lebanon can be considered hazardous and may pose a significant health risk to the Lebanese population, especially children (Daou et al. 2020; Milićević et al. 2021). These results are consistent with the risk assessment in the present study and suggest that, in most cases, the presence of AFM1 in milk and dairy products carries a carcinogenic risk for children.

A study in India found that health risk assessment showed that the daily intake of AFM1 was higher than the established tolerable daily limit for both adults and children (HI > 1), indicating a high potential health risk for consumers, consistent with the results of the present four‐season dataset (Hattimare et al. 2022).

In a study in Serbia (2017–2019), researchers investigated the exposure and health risk of AFM1 from milk and milk‐based products among children. AFM1 was widely detected in 2%–79% of the 3404 samples, with pasteurized and UHT milk showing the highest incidence (79%) and mean concentration (22.34–0.018 ng/kg). Exposure analysis revealed that toddlers (1–3 years old) had the highest EDI, up to 0.193 ng/kg bw/day, due to their greater milk consumption relative to body weight. However, risk characterization using the MOE showed values >10000, and the estimated risk of HCC was negligible, indicating a low overall health risk for all evaluated age groups (Milićević et al. 2021). The AFM1 exposure in Serbia is characterized by low concentrations that translate to a negligible health risk for children, suggesting effective regulatory controls. The present four‐season dataset indicated elevated AFM1 exposure levels for the paediatric population, suggesting medium non‐carcinogenic and potential carcinogenic risks. These findings highlight the need for seasonally informed mitigation strategies.

It appears necessary to protect consumer health and to increase farmers’ and livestock owners’ awareness of proper livestock feed storage methods. Regulatory bodies should also enforce milk safety‐related regulations and supervise their implementation to help improve the health level of milk consumers in Arak province.

Collectively, these findings suggest that AFM1 contamination in Markazi Province follows a clear seasonal pattern driven by climatic conditions and feed contamination, with raw and pasteurized milk posing the highest risk, particularly for children. These results align with broader trends observed in other Iranian provinces and highlight the need for region‐specific monitoring strategies.

To evaluate the robustness of the MOE results, a brief sensitivity assessment was performed. MOE values were calculated under both median (P50) and high‐end (P95) exposure scenarios. As expected, MOE values decreased under the P95 scenario due to higher estimated intake, indicating a more conservative risk characterization. We also examined the effect of varying milk consumption within a plausible range around the national per‐capita value. Although these alternative assumptions resulted in numerical changes in MOE, the overall interpretation remained unchanged: High‐end exposure scenarios consistently produced lower MOE values, but the qualitative conclusion regarding potential health concerns did not differ. This demonstrates that the MOE‐based conclusions are robust to reasonable variations in exposure assumptions.

The interpretation of this study should be viewed with consideration of several limitations. First, the regional scope of sampling restricts the generalizability of the findings. Second, the dataset represents a single year of monitoring, which limits the ability to capture longer term trends. Third, no direct measurements of climatic conditions or feed contamination were available, preventing a more detailed explanation of the observed seasonal patterns. Fourth, uncertainties remain in high‐percentile consumption assumptions due to the absence of age‐specific national dietary data. Despite these limitations, the results underscore the need for improved dietary datasets and expanded monitoring programmes in Iran.

## 5 Conclusion

This study identified clear differences in AFM1 contamination across milk types and seasons in Markazi Province, with raw milk showing the highest levels and UHT milk the lowest. Seasonal variation was evident, with higher AFM1 concentrations generally observed in summer, underscoring the influence of environmental conditions and the need for intensified monitoring during high‐risk periods. Exposure assessment indicated that high‐end scenarios (P95) produced the lowest MOE values, highlighting children—especially consumers of raw and pasteurized milk—as the most vulnerable group. These findings emphasize the importance of effective feed management and contamination‐control strategies across the dairy supply chain. Overall, controlling AFM1 contamination in milk and dairy products should be considered a serious priority in Iran's food‐safety policies.

## Author Contributions

**Pedram Hashemi**: investigation, sample collection and analysis. **Mohammadhosein Movassaghghazani**: supervision, project administration, exposure assessment, writing, reviewing and editing. **Milica Zekovic**: writing, reviewing and editing. All authors have read and agreed to the published version of the manuscript.

## Funding

The authors have nothing to report.

## Ethics Statement

The authors confirm that the ethical policies of the journal, as noted on the journal's author guidelines page, have been adhered to, and the appropriate ethical review committee approval has been received. The study was approved by the Ethics Committee of the Islamic Azad University‐Tabriz branch (Approval ID: IRIAU.TABRIZ.REC.1403.484).

## Conflicts of Interest

The authors declare no conflicts of interest.

## Data Availability

Data are available on request from the authors.
